# Effects of group counseling on height dissatisfaction and attentional bias in adolescence

**DOI:** 10.3389/fpsyg.2026.1892947

**Published:** 2026-07-21

**Authors:** Shan Yi

**Affiliations:** 1 School of Education Science, Hunan Normal University, Changsha, China; 2 School of Marxism, Hunan Normal University, Changsha, China

**Keywords:** adolescence, attentional bias, dot-probe task, group counseling, height dissatisfaction

## Abstract

Height dissatisfaction is a prevalent negative physical self-problem in adolescence. Existing group interventions predominantly focus on improving explicit body cognition while neglecting implicit attentional processing. This study adopted a pre-test and post-test randomized controlled design to explore the intervention effects and cognitive mechanisms of group counseling on adolescent height dissatisfaction. Adolescents with height dissatisfaction received six sessions of standardized group counseling. Explicit height dissatisfaction was measured by self-report scales, and the subliminal dot-probe paradigm was applied to assess implicit attentional bias. The results revealed group counseling effectively alleviated adolescents’ explicit height dissatisfaction, whereas it produced only limited effects on modifying implicit attentional bias. This study compensates for the limitations of single explicit evaluation methods, provides empirical evidence for the cognitive mechanism of group intervention for adolescent height dissatisfaction, and offers practical implications for optimizing future intervention programs.

## Introduction

Body dissatisfaction can be defined as an attitudinal construct that reflects individuals’ negative evaluations of their overall body, as well as specific aspects of one’s body such as body size, shape, muscularity, and weight (Cash and Deagle, 1997; Chen et al., 2006; Grogan, 2021). Body dissatisfaction is closely linked to a range of negative health consequences and behaviors. For example, in adolescence it is associated with later anxiety and depression episodes (Barnes et al., 2020; Bornioli et al., 2021), as well as with risk factors for self-injurious thoughts and behaviors (Perniciaro et al., 2024; Ren et al., 2024), drug-use (Rocha et al., 2022), disordered eating behaviors (Bornioli et al., 2019; Yang et al., 2022), and smartphone addiction (Liu et al., 2023). For these reasons, body dissatisfaction is considered a serious public health concern.

Height dissatisfaction has been identified as an important dimension of negative physical self among Chinese adolescents, with previous studies suggesting that concerns about stature are particularly salient during adolescence (Chen et al., 2006; Sun, 2017). Unlike body weight or body shape, height is largely determined by genetic and developmental factors and is difficult to modify after puberty. Consequently, height dissatisfaction may persist once established and has become a prevalent psychological concern among Chinese adolescents, highlighting the need to better understand its underlying cognitive mechanisms and develop targeted intervention strategies.

Cognitive schema theory posits that negative body self-schema drives abnormal attentional processing, which serves as a core mechanism maintaining persistent body dissatisfaction (Williamson et al., 2004; Rodgers and DuBois, 2016; Cass et al., 2020). Individuals with negative physical self automatically prioritize schema-congruent negative body information, exhibiting typical attentional vigilance toward negative body cues and difficulty in attentional disengagement from negative stimuli (Vitousek and Hollon, 1990; Jackman et al., 1995). Empirical studies have confirmed that high body dissatisfaction is closely correlated with implicit attentional bias toward negative body-related information (Rodgers and DuBois, 2016; Jiang and Vartanian, 2018). Correspondingly, adolescents with height dissatisfaction tend to exhibit automatic attentional bias toward height-related negative stimuli, representing a stable implicit cognitive characteristic of height dissatisfaction. However, previous intervention studies have primarily focused on improving explicit body dissatisfaction, with little attention paid to changes in implicit attentional processing. Consequently, it remains unclear whether conventional cognitive-behavioral group counseling can effectively modify implicit height-related attentional bias.

Group cognitive-behavioral counseling has become a mainstream and effective intervention for adolescent body dissatisfaction. Common intervention forms include cognitive behavioral group therapy, dissonance-based body image intervention, and pubertal health group counseling, all of which can effectively correct adolescents’ explicit irrational body cognition and reduce negative body evaluation (Stice et al., 2019; Ciao et al., 2021; Maas et al., 2023; Pullmer et al., 2023; Nazmi et al., 2024). Nearly all existing relevant studies rely solely on explicit self-report questionnaires to evaluate intervention effects, while few adopt behavioral experimental paradigms to track changes in implicit body-related attentional bias before and after interventions. This single evaluation method fails to distinguish the differential malleability of explicit conscious cognition and implicit automatic cognitive processing, resulting in an incomplete understanding of the underlying intervention mechanisms.

The dot-probe paradigm is the most classic and widely adopted experimental task for measuring body-related implicit attentional bias (Glauert et al., 2010; House et al., 2023). Nevertheless, accumulating methodological evidence indicates that the dot-probe task has relatively low temporal reliability and limited sensitivity to subtle changes in automatic attentional tendencies (Schmukle, 2005; Price et al., 2015; Parsons et al., 2019). Meanwhile, existing attentional bias modification interventions show unstable and limited effects on reshaping implicit cognitive bias (Loughnan et al., 2015; Engel et al., 2019; House et al., 2022, 2023). These findings imply that implicit schematic cognitive processing has high stability and cognitive inertia, and short-term psychological interventions may struggle to effectively alter ingrained implicit attentional patterns, which may lead to a dissociation between explicit and implicit intervention outcomes. However, this speculation lacks empirical verification in the field of adolescent height dissatisfaction intervention. In the present study, the dot-probe paradigm was employed solely as a behavioral assessment tool to evaluate changes in implicit attentional bias before and after cognitive-behavioral group counseling, rather than as an attentional bias modification intervention.

The present study adopted a pre-test-post-test randomized controlled design to explore the intervention effects of six-session cognitive-behavioral group counseling on explicit height dissatisfaction and implicit height-related attentional bias among junior high school students with height dissatisfaction. Explicit negative physical self was measured via standardized questionnaires, while the subliminal dot-probe paradigm was employed to assess two core implicit attentional indicators: attentional vigilance and attentional disengagement toward height-related stimuli. This study aimed to examine whether short-term group counseling can simultaneously improve adolescents’ explicit height dissatisfaction and reshape their implicit attentional bias, and further explore the differential malleability of explicit and implicit body cognition. The results of this study can provide empirical evidence for explaining the cognitive mechanism of group counseling intervention, and offer theoretical and practical references for the prevention and clinical intervention of adolescents’ height-specific negative physical self.

## Materials and methods

### Participants

Participants were 32 adolescent students, from the Affiliated School of Inner Mongolia Normal University. Inclusion criteria were: 12–15 years old; in junior high school (grades 7–9); self-identification as having body image and a score greater than 2.5 on the Shortness Concern subscale of the Negative Physical Self Scale (NPSS-S; Chen et al., 2006). In addition, were considered to be the best judges of their need for assistance (Heinicke et al., 2007).

Exclusion criteria were: with acute suicidality, severe depressive symptoms, body mass index (BMI) > 25 kg/m^2^, autism spectrum disorder (ASD), IQ < 70, schizophrenia or other psychotic disorders, uncorrected vision impairments or currently having specialist treatment for an eating disorder or another psychiatric disorder.

These participants were randomly assigned to the intervention group (*n* = 16) and the control group (*n* = 16). Full informed consent was obtained from both participants and their guardians. The study was approved by the ethics committee of the Inner Mongolia Normal University.

### Materials

#### Shortness concern subscale of the negative physical self scale

Body dissatisfaction was assessed with the Shortness Concern subscale of the Negative Physical Self Scale (NPSS-S; Chen et al., 2006). The NPSS-S measures dissatisfaction with one’s height. This subscale was selected as it has been shown to yield valid and reliable scores in adolescent and young adult samples (Ly et al., 2019; Wang et al., 2022). The NPSS-S contained 11 items assessing three dimensions (affective-cognitive, projective, and behavioral regulation) related to height dissatisfaction. Each item is rated on a five-point Likert-type scale ranging from 0 ( = “never”) to 4 ( = “always”). Item scores were summed, with a higher score indicating greater height dissatisfaction. In the present study, Cronbach’s alpha for the NPSS-S was 0.81.

#### Body mass index

Body mass index (BMI) was calculated for each participant by recording their self-reported weight (in kg) and dividing it by the square of the height (cm) and expressed in units of kg/m^2^. Although self-reported height and weight in adolescent students tend to be reliable and valid, there is a tendency to over-estimate height and under-estimate weight (Himes et al., 2005). As the present study examined change across time within individuals, this potential bias was not considered problematic (Heinicke et al., 2007).

#### Group member self-rating scale

The Group Member Self-Rating Scale was used to evaluate the individual’s level of participation during the group intervention (Fan, 1996). The scale consisted of 20 items, rated on a 5-point Likert scale ranging from 1 (never) to 5 (always), with higher total scores indicating greater levels of individual participation in the group.

#### Height-related words

The height-related words were selected from a previous study (Meng, 2013). A total of 16 short-related words (e.g., dwarf) and 16 tall-related words (e.g., giant) were selected as experimental stimuli. All neutral words consisted of household appliance and furniture terms (e.g., battery), with a total of 64 items. The neutral words were matched to the height-related words in word length (number of Chinese characters). Three types of word pairs were constructed: short-neutral (S–N), tall-neutral (T–N), and neutral-neutral (N–N). An additional 24 neutral words unrelated to the experimental materials (e.g., transportation-related words such as “airplane” and “tank”) were selected as practice stimuli.

Word frequency and familiarity were rated by three middle school Chinese language teachers on 5-point scales, confirming that the three-word categories were well matched on these dimensions. Additional selection criteria ensured that all words were age-appropriate and commonly used by junior high school students.

#### Dot-probe task

**FIGURE 1:**
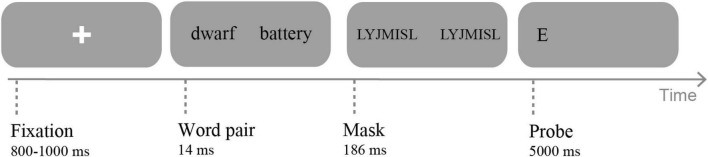
Illustration of the experimental procedure. Each trial consisted of a fixation cross, a height-related word pair, a mask, and a probe presented sequentially. Participants were instructed to identify the probe letter as quickly and accurately as possible.

Attentional bias was measured using a dot-probe task. Participants were seated at about 60 cm viewing distance in front of a monitor. No chin rest was used. At the beginning of the dot-probe task, a fixation cross was randomly presented in the center of the screen for 800–1000 ms (see Figure 1). Subsequently, one type of word pair word pairs (S–N, T–N, and N–N) appeared for 14 ms, with one word presented on the right half and one on the left half of the screen with a distance of 60 cm. Following the presentation of the word pairs, masking stimuli was shown for 186 ms at the location of the word pairs. Masking stimuli consisted of a string of seven random consonants (e.g., “LYJMISL”) matched to the words in length. Finally, a target probe, either “E” or “F,” was randomly presented on either the left or right side of the screen. The probe location was consistent with the location where the preceding word had appeared. Trials were classified as congruent when the height-related word and the probe appeared on the same side, and incongruent when they appeared on opposite sides. Participants were instructed to react to the target probe as quickly and accurately as possible by pressing the “E” key on the keyboard for “E” and the “F” key for “F.”

The experiment comprised two blocks. Each block contained 32 trials per condition (S–N congruent, S–N incongruent, T–N congruent, T–N incongruent, and N–N), with the order of trials randomized within each block.

### Group counseling intervention program

The complete intervention protocol is presented in Supplementary Appendix to facilitate replication of the study. A structured group intervention program was implemented among junior high school students. The intervention consisted of six weekly 50-minute group sessions delivered over a six-week period, facilitated by a trained group leader using a standardized manual. The program was organized into three sequential phases: an initial group formation phase, an in-depth self-exploration phase focused on body image and height acceptance, and a planned termination phase.

The first session aimed to build rapport, reduce initial awkwardness, enhance group belongingness, introduce program goals and confidentiality guidelines, and establish group norms. Icebreaking and bonding activities were used to create a safe and supportive climate for subsequent self-disclosure and exploration.

The four middle sessions constituted the core intervention phase. The second session guided participants to examine their actual and ideal self-concepts, identify discrepancies related to physical characteristics such as height, and develop a balanced and realistic framework for self-evaluation. The third session focused on the impact of physical appearance on self-identity, encouraged students to recognize personal strengths beyond physical features, and fostered self-compassion and acceptance toward their natural height and body. The fourth session broadened students’ understanding of health and personal worth, reduced over-reliance on height as a basis for self-evaluation, and strengthened peer support through collaborative tasks and positive feedback. The fifth session helped students acknowledge universal physical limitations, normalize concerns about height, challenge irrational beliefs about body image, practice self-acceptance, and receive advance notice of program closure to prepare for emotional transition.

The final session served as the termination phase. Its primary objectives were to consolidate intervention effects, reflect on personal growth related to height acceptance and self-esteem, address feelings of separation, and collect participant feedback. The session included relaxation exercises, sincere sharing, peer affirmation, and a structured summary by the facilitator.

This six-week, weekly format was chosen to maintain student engagement while fitting within regular school schedules. Grounded in cognitive behavioral principles, positive psychology, and group dynamics, the program provided a safe, supportive, and confidential environment. Through psychoeducational input, interactive activities, peer discussion, and guided reflection, the intervention aimed to reduce height-related body dissatisfaction, improve body image, and enhance self-acceptance among junior high school students.

### Procedure

The present study comprised three sequential phases: preparation, intervention, and assessment.

Preparation (baseline/pre-test): Eligible participants were randomly assigned to either the intervention group or the waiting-list control group by group facilitators using a computer-generated random allocation sequence. All participants in both groups completed pre-intervention assessments, consisting of the NPSS-S scale and a dot-probe task.

Intervention: Participants in the intervention group received a 6-week, structured group counseling intervention specifically designed to reduce height-related body dissatisfaction. Participants in the waiting-list control group received no group intervention during the corresponding intervention period.

Assessment (post-test): One week after the completion of the intervention period, all participants in both groups completed post-intervention assessments, including the NPSS-S scale and the dot-probe task. Additionally, participants in the intervention group completed the Group Member Self-Rating Scale to assess their subjective experiences, engagement, and perceived benefits from the group program.

### Data analysis

All statistical analyses were conducted using Rstudio software. For the dot-probe task, reaction times shorter than 200 ms or longer than 2000 ms were firstly excluded, and trials with reaction times beyond ± 3 standard deviations of the individual mean were further eliminated as outliers. Attentional bias indices were calculated following Koster et al. (2004). The vigilance score was computed as mean reaction time of neutral trials minus mean reaction time of congruent trials, reflecting facilitated attention toward negative height-related stimuli. The disengagement score was calculated as mean reaction time of incongruent trials minus mean reaction time of neutral trials, indexing difficulty in attentional disengagement from negative stimuli.

In the current study, four types of attentional bias indices were derived accordingly, namely S-attentional vigilance, S-attentional disengagement, T-attentional vigilance, and T-attentional disengagement. Specifically, S-attentional vigilance referred to facilitated attention toward short-height-related words; S-attentional disengagement indicated the difficulty in disengaging attention from short-height-related words; T-attentional vigilance reflected facilitated attention toward tall-height-related words, whereas T-attentional disengagement indexed the difficulty in attentional disengagement from tall-height-related words.

Repeated-measures ANOVA was adopted to examine group, time and condition effects on scale scores and dot-probe reaction times. One-sample *t*-tests were used to verify baseline attentional bias. Independent and paired-samples *t*-tests were applied for simple effect comparisons and pre-post changes.

## Results

### Sample characteristics

A total of 32 junior high school students aged 12–15 years were initially recruited for the study. Two participants dropped out of the study: one withdrew midway through the group counseling sessions, and the other failed to complete the post-test of the dot-probe task. This yielded a final valid sample of 30 participants. The participants were randomly assigned to either the intervention group or the control group, with 15 students in each group. The baseline demographic and anthropometric characteristics of the participants, including age, height, weight, and BMI, were comparable between the two groups (*p* > 0.05 for all comparisons). Detailed information is shown in Table 1.

**TABLE 1:** General information of junior high school students.

Measure	Intervention group	Control group	*t*	*p*	Cohen’s d
N	15	15			
Age (years)	13.87 (0.83)	13.53 (0.99)	−0.99	0.33	−0.36
Height (cm)	160.40 (3.94)	163.40 (6.91)	1.46	0.16	0.53
Weight (kg)	50.27 (12.09)	49.37 (12.08)	−0.21	0.83	−0.08
BMI (kg/m^2^)	19.50 (4.49)	18.47 (4.05)	−0.66	0.51	−0.24

### NPSS-S scale outcomes

Table 2 presents descriptive and inferential statistics of pre- and post-test scores for the two groups. A 2 (group: intervention vs. control) × 2 (time: pre-test vs. post-test) repeated-measures ANOVA was performed to examine the changes in questionnaire scale scores. The results showed that the main effects of group [*F* (1, 28) = 6.37, *p* = 0.02, η_*p*_^2^ = 0.19] and time [*F* (1, 28) = 19.86, *p* < 0.001, η_*p*_^2^ = 0.42] was significant. Importantly, a significant group × time interaction effect was observed [*F* (1, 28) = 5.41, *p* = 0.027, η_*p*_^2^ = 0.16].

**TABLE 2:** Means, standard deviations, between-group and within-group comparisons of NPSS-S scale measures at pre-test and post-test.

Measure	Intervention group	Control group	*t*_(28)_	*p*	Cohen’s d
Pre-test	2.98 (0.38)	3.12 (0.34)	1.09	0.29	0.25
Post-test	2.26 (0.69)	2.90 (0.56)	2.76*	0.01	1.12
*t*_(28)_	−4.80***	−1.51			
*p*	< 0.001	0.14			
*Cohen’s d*	−1.26	−0.40			

Values are presented as mean (standard deviation). *p < 0.05, **p < 0.001, ***p < 0.001. Degrees of freedom for *t*-tests = 28. Cohen’s *d* indicates effect size.

Further simple effect analysis revealed that the intervention group showed a significant decrease in scale scores from pre-test to post-test (*t*_28_ = −4.80, *p* < 0.001, Cohen’s *d* = −1.26), while no significant pre-post change was found in the control group (*t*_28_ = −1.51, *p* = 0.14, Cohen’s *d* = −0.40). At post-test, the scale scores of the intervention group were significantly lower than those of the control group (t_28_ = 2.76, *p* = 0.01, Cohen’s *d* = 1.12).

### Dot-probe task outcomes

Accuracy rates exceeded 90% across all experimental conditions and thus accuracy data were excluded from further statistical analysis.

#### Reaction times

Table 3 presents the means and standard deviations of reaction times across all conditions in the dot-probe task. For reaction times, a 2 (group: intervention vs. control) × 2 (time: pre-test vs. post-test) × 5 (condition: S-N congruent, S-N incongruent, T-N congruent, T-N incongruent vs. N-N neutral) repeated-measures ANOVA was performed. The results revealed a significant main effect of condition, *F* (3.54, 99.11) = 2.64, *p* = 0.044, η_*p*_^2^ = 0.086. No significant main effects of group [F (1, 28) = 0.02, *p* = 0.896, η_*p*_^2^ = 0.001] or time [*F* (1, 28) = 0.19, *p* = 0.664, η_*p*_^2^ = 0.007] were observed. Critically, the group × time interaction, which was the primary test of the intervention effect, was not significant [*F* (1, 28) = 1.51, *p* = 0.229, η_*p*_^2^ = 0.051], indicating that the intervention did not produce a significantly different change in reaction times compared with the control condition over the study period. The two-way interactions of group × condition *F* (3.54, 99.11) = 0.86, *p* = 0.48, η_*p*_^2^ = 0.030] and time × condition [*F* (3.13, 87.57) = 2.36, *p* = 0.075, η_*p*_^2^ = 0.078], as well as the three-way interaction [*F* (3.13, 87.57) = 1.159, *p* = 0.331, η_*p*_^2^ = 0.040], did not reach the conventional significance level.

**TABLE 3:** Means and standard deviations of reaction times across all experimental conditions in the dot-probe task.

	Intervention group		Control group	
Condition	Pre-test	Post-test	Pre-test	Post-test
S-N congruent	488.64 (117.30)	479.04 (57.79)	467.25 (65.36)	491.11 (67.87)
S-N incongruent	513.31 (138.18)	471.14 (61.50)	486.29 (69.81)	504.26 (93.26)
T-N congruent	508.77 (131.73)	458.45 (54.61)	485.38 (59.90)	494.48 (71.74)
T-N incongruent	508.67 (127.07)	463.87 (51.40)	487.38 (83.97)	504.30 (85.36)
N-N neutral	515.66 (142.90)	467.45 (57.55)	479.17 (71.06)	503.84 (86.92)

Values are presented as mean (standard deviation). Reaction times are reported in milliseconds.

#### Attentional bias

Table 4 presents the means and standard deviations of attentional bias across all conditions in the dot-probe task. Four attentional biases, including S-attentional vigilance, S-attentional disengagement, T-attentional vigilance, and T-attentional disengagement, were calculated. To confirm the existence of baseline attentional bias, one-sample *t*-tests (test value = 0) were conducted separately for the intervention group and the control group on all four indices, yielding eight baseline comparisons in total. A significant baseline S-attentional vigilances for intervention (*t*_14_ = 2.57, *p* = 0.02, *Cohen’s d* = 0.66) and control group (*t*_14_ = 2.97, *p* = 0.01, *Cohen’s d* = 0.77) were found. By contrast, no significant baseline bias was observed for S-attentional disengagement, T-attentional vigilance, and T-attentional disengagement in either group (all *p* > 0.05). These preliminary findings indicated that junior high school students with height dissatisfaction exhibited a specific attentional vigilance tendency toward height-negative stimuli, confirming a targeted attentional bias for height-related negative words at baseline.

**TABLE 4:** Means and standard deviations of attention bias scores across all experimental conditions in the dot-probe task.

	Intervention group		Control group	
Attention bias	Pre-test	Post-test	Pre-test	Post-test
S-attentional vigilance	27.01 (40.74)	−11.59 (38.94)	11.92 (15.54)	12.73 (36.07)
S-attentional disengagement	−2.34 (30.58)	3.70 (40.01)	7.12 (28.73)	0.42 (28.78)
T-attentional vigilance	6.88 (32.37)	8.99 (29.30)	−6.21 (25.60)	9.36 (27.60)
T-attentional disengagement	−6.99 (34.99)	−3.58 (30.45)	8.21 (34.03)	0.46 (34.81)

**FIGURE 2:**
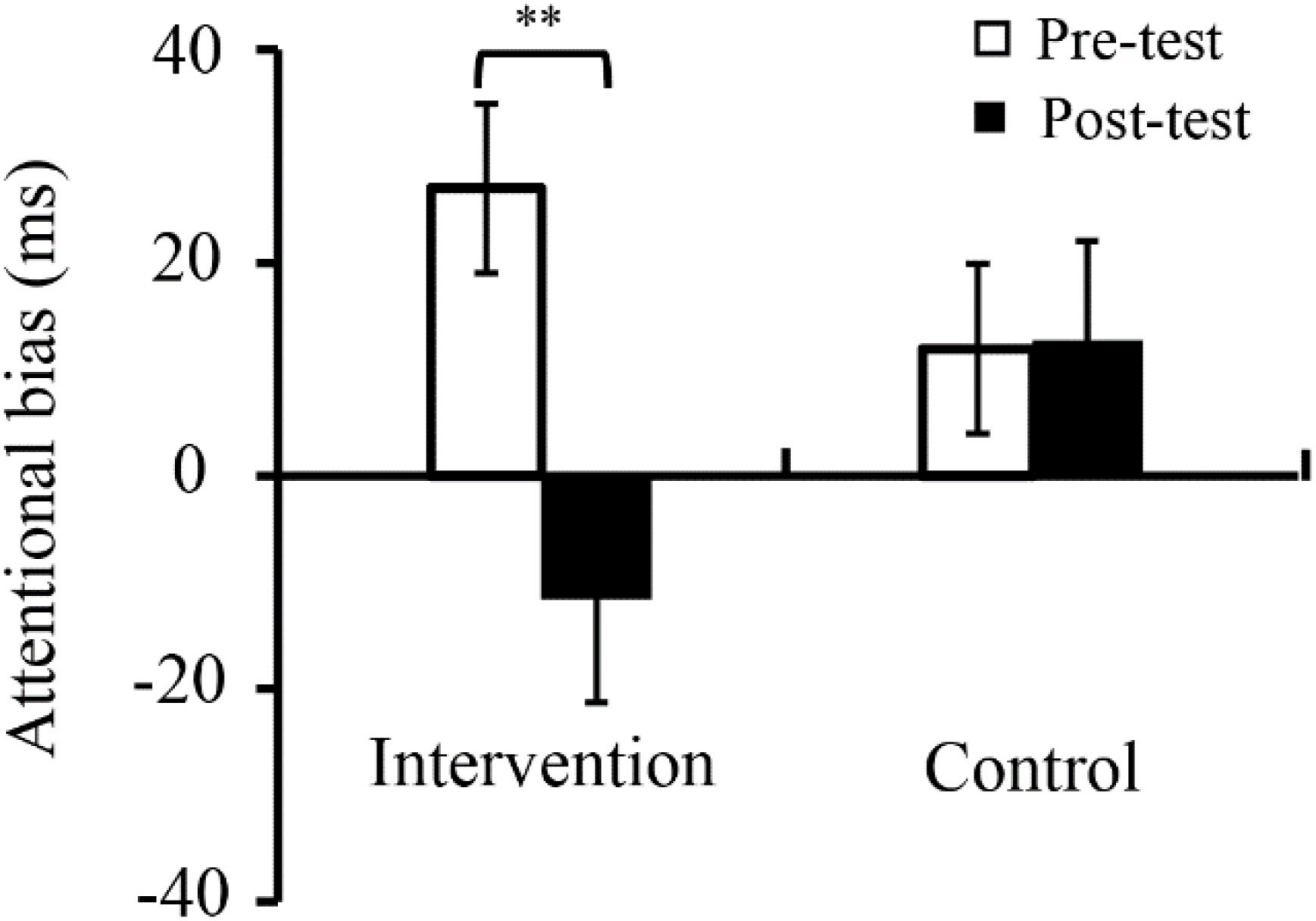
Pre-test and post-test attentional bias (S-attentional vigilances) for the intervention and control groups. Error bars represent standard error of the mean. Positive values indicate attentional bias toward negative height-related stimuli. **Represent *p* < 0.01.

Figure 2 shows the pre-test and post-test attentional bias (S-attentional vigilances) for the intervention and control groups. S-attentional vigilances was only selected to explore the intervention effect of group counseling on attentional bias. A 2 (group: intervention vs. control) × 2 (time: pre-test vs. post-test) repeated-measures ANOVA was performed. The results showed no significant main effect of group [*F* (1, 28) = 0.28, *p* = 0.60, η_*p*_^2^ = 0.01]. A significant main effect of time was observed [*F* (1, 28) = 4.36, *p* = 0.046, η_*p*_^2^ = 0.14], as well as a significant group × time interaction [*F* (1, 28) = 4.74, *p* = 0.038, η_*p*_^2^ = 0.15].

Further simple effect analysis revealed that the intervention group showed a significant decreased attentional bias performance from pre-test to post-test (*t*_28_ = −3.02, *p* = 0.005, *Cohen’s d* = −0.79), while no significant pre-post change was found in the control group (*t*_28_ = 0.06, *p* = 0.95, *Cohen’s d* = 0.02). However, at post-test, no significant difference in attentional bias was found between the intervention group and the control group (*t*_28_ = 1.77, *p* = 0.087, *Cohen’s d* = 0.50).

### Quantitative outcomes of the group member self-rating scale

Fifteen participants in the intervention group completed the Group Member Self-Rating Scale after the fifth session of group counseling. The scale was scored on a 1–5 Likert scale. The mean scores of participants ranged from 3.30 to 4.75, indicating that members were generally satisfied with their performance in group activities. They actively engaged in the sessions, enjoyed the group experience, and embraced the core concepts delivered by the counseling. These findings demonstrated that the group counseling at this stage achieved favorable outcomes.

## Discussion

The present study aimed to explore the effects of cognitive-behavioral group counseling on explicit height dissatisfaction and implicit attentional bias among junior high school students with height dissatisfaction. Thirty adolescents were randomly assigned to either an intervention group or a control group, with explicit negative physical self-measured by questionnaire scales and implicit attentional bias assessed via the subliminal dot-probe paradigm. Overall, the results revealed a clear dissociation between explicit and implicit intervention outcomes: group counseling effectively reduced explicit height dissatisfaction but exerted only limited influence on implicit attentional bias toward height-related negative stimuli.

Junior high school students with height dissatisfaction exhibited a significant attentional bias toward height-related negative words at the pre-test stage, which was consistent with previous research (Cho and Lee, 2013; Cai et al., 2020). According to cognitive schema theory, individuals with negative physical self tend to automatically activate body-schema processing when exposed to height and body shape information, prioritizing attention to negative schema-congruent information and showing difficulty in disengaging from negative cues (Vitousek and Hollon, 1990; Jackman et al., 1995). In the current study, both the intervention and control groups scored above 2.5 on the NPSS-S scale and showed similar reaction times across all dot-probe conditions, indicating good homogeneity in both explicit self-evaluation and implicit attentional bias at baseline. This finding is consistent with the typical cognitive processing pattern observed in individuals with body dissatisfaction, who automatically allocate attention to negative body-related information (Mendoza-Medialdea et al., 2023).

Following six sessions of group counseling, the intervention group showed a significant reduction in attentional bias from pre-test to post-test, but post-test intergroup comparisons failed to reach statistical significance. Collectively, these findings suggested that the current short-term group counseling protocol exerted only a constrained effect on reshaping implicit attentional bias among adolescents with height dissatisfaction. Several underlying reasons may account for such limited intervention efficacy. First, subliminal attentional processing operates at the pre-conscious level and is inherently resistant to modification via brief psychological intervention (Zhang et al., 2024). Second, accumulating methodological studies have indicated that the dot-probe paradigm suffers from relatively low temporal reliability and limited predictive validity, restricting its sensitivity to detect subtle alterations in automatic attentional tendencies (Schmukle, 2005; Price et al., 2015; Parsons et al., 2019). Third, body dissatisfaction and attentional bias do not conform to a simple causal pathway, rendering it challenging for conventional short-term group intervention to reconstruct stable automatic cognitive patterns (Stott et al., 2021; Mendoza-Medialdea et al., 2023). Additionally, pervasive social media aesthetic norms continuously reinforce adolescents’ internalization of ideal height and body standards, further consolidating implicit cognitive biases and weakening the malleability of automatic processing (Mazzeo et al., 2024).

Different from the limited effect on implicit attentional bias, group counseling achieved a significant improvement in explicit height dissatisfaction. Post-test scores on the negative physical self-scale in the intervention group were significantly lower than those in the control group, suggesting that the cognitive-behavioral group counseling effectively alleviated adolescents’ explicit height dissatisfaction. This finding supports existing meta-analytic evidence that cognitive behavioral intervention can correct irrational body-related cognition, reduce overemphasis on physical appearance, and enhance body acceptance (Zamiri-Miandoab et al., 2021). Notably, the current intervention prioritizes the adaptive value of physical functionality above superficial aesthetic appearance, consistent with existing literature that adolescent body image evaluation is determined by perceived physical competence and health status, in addition to physical attractiveness (Abbott and Barber, 2010; Swami et al., 2010). Despite significant post-intervention reductions in explicit scale scores, participants in the intervention group still maintained scores above the 2.5 threshold, remaining categorized as individuals with height dissatisfaction. Supplementary evidence from the Group member self-rating scale further validated that participant engaged actively in group interactions, subjectively perceived meaningful improvements in body-related cognition, and acknowledged the clinical value of the intervention program.

Despite the favorable effects observed in explicit body self-perception, the present intervention demonstrated limited capacity to modify ingrained implicit attentional patterns. Several methodological and practical limitations of this study should be acknowledged. First, the intervention content lacked comprehensiveness. Future research should systematically explore the factors contributing to adolescents’ negative physical self to develop more targeted and individualized intervention protocols. Second, the intervention dosage was relatively insufficient, as only six sessions may be inadequate to alter stable implicit cognitive schemas. Future studies are recommended to adopt medium-to-long-term intervention with no fewer than 12 sessions. Third, the current evaluation system relied merely on quantitative scales and behavioral experimental tasks, without incorporating in-depth qualitative interviews, which restricted a comprehensive understanding of participants’ subjective experiences and psychological needs. Fourth, the study lacks long-term follow-up assessments. Although the intervention effectively improved explicit height dissatisfaction immediately after the program, it remains unclear whether these improvements can be maintained over time. Future studies should incorporate delayed follow-up assessments (e.g., 3-, 6-, or 12-month follow-ups) to evaluate the durability of intervention effects and further examine the long-term trajectories of both explicit body dissatisfaction and implicit attentional bias.

In summary, the current study systematically explored the effects of cognitive-behavioral group counseling on explicit height dissatisfaction and implicit attentional bias among junior high school students. The results confirmed a clear dissociation of intervention effects between explicit and implicit cognitive levels: short-term group counseling effectively ameliorated explicit height dissatisfaction and restructured conscious body-related self-cognition, yet failed to induce substantial changes in subliminal implicit attentional bias. Such dissociation implies that explicit self-evaluation is more amenable to psychological intervention, whereas implicit schematic cognitive processing possesses high stability and cognitive inertia. The present findings provide empirical evidence and theoretical implications for the preventive and therapeutic intervention of early adolescents’ negative physical self and height dissatisfaction. Meanwhile, this study underscores the necessity of extending intervention duration, diversifying intervention components, and adopting multi-dimensional mixed-method evaluation in future relevant research.

## Data Availability

The raw data supporting the conclusions of this article will be made available by the author, without undue reservation.
