# Supplementary material for: Effects of group counseling on height dissatisfaction and attentional bias in adolescence
Source: Front Psychol. 2026 Jul 21;17:1892947. doi: 10.3389/fpsyg.2026.1892947 (PMC13433440; doi:10.3389/fpsyg.2026.1892947)
Supplement: Supplementary file 1 [file Table_1.docx]

## Appendix A. Six-Session Cognitive-Behavioral Group Counseling Program

Table A1. Overview of the cognitive-behavioral group counseling program.

| Session | Objectives | Group Activities |
| --- | --- | --- |
| 1. Building Group Cohesion | • Establish rapport among members.  • Enhance group cohesion and participation.  • Introduce group rules and confidentiality. | • Facilitator introduction and orientation  • Ice-breaking activity ('Big Wind Blows')  • Snowball introduction  • Group agreement  • Expectations card  • Group reflection |
| 2. Knowing Myself | • Increase self-awareness.  • Compare the real self with the ideal self.  • Develop healthy self-evaluation. | • Name-chain warm-up  • Self-portrait exercise  • Feedback from peers  • Group discussion and reflection |
| 3. Learning to Accept Myself | • Explore the influence of body image on self-concept. • Recognize personal strengths.  • Promote self-acceptance. | • Mirror reflection exercise  • Circle of appreciation  • Sharing experiences of receiving care and acceptance  • Group reflection |
| 4. Recognizing My Strengths | • Promote a healthy understanding of self-worth.  • Identify individual strengths and valued qualities. | • Warm-up ('No I' game)  • Cooperative task  • Positive feedback exercise  • Group reflection |
| 5. Accepting Imperfections | • Recognize personal limitations.  • Develop self-acceptance.  • Practice adaptive coping strategies. | • Personal growth exercise  • 'My AD' exercise  • Group discussion  • Preparation for group termination |
| 6. Looking Ahead | • Review the intervention.  • Consolidate gains.  • Facilitate healthy group closure. | • Gentle stretching warm-up  • Positive feedback messages ('Angel Notes')  • Sharing experiences and suggestions  • Facilitator summary |
